# Aspiration pneumonia associated with achalasia

**DOI:** 10.1002/jgf2.524

**Published:** 2022-02-03

**Authors:** Yuri Ono, Hiroyuki Kobayashi, Kazuya Nagasaki, Hiroyuki Ariga, Junya Kashimura

**Affiliations:** ^1^ Department of Internal Medicine Kamisu Saiseikai Hospital University of Tsukuba Ibaraki Japan; ^2^ Department of Internal Medicine Mito Kyodo General Hospital University of Tsukuba Ibaraki Japan; ^3^ Department of Gastroenterology Mito Kyodo General Hospital University of Tsukuba Ibaraki Japan

**Keywords:** achalasia, aspiration pneumonia, dysphagia

## Abstract

In this case, a chest computed tomography scan of a young patient with pneumonia revealed esophageal obstruction, which led to the diagnosis of aspiration pneumonia due to achalasia. This report highlights achalasia and other gastrointestinal obstructions as one of the causes of aspiration pneumonia.

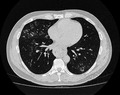

A 33‐year‐old man with chronic cough presented with worsening cough associated with fever, more frequently in the morning. Although he had been administered antihistamines, inhaled corticosteroids, and antibiotics, his chronic cough persisted for 2 months without improvement. The patient had no other remarkable medical history. Moreover, he had no history of medication use or heavy drinking. Physical examination showed bilateral coarse crackles. Chest radiography demonstrated ground‐glass shadows in both lungs with no other findings (Figure 1). Thoracic computed tomography revealed small centrilobular nodular shadow in both lungs (Figure 2A). Dilation of a food‐filled esophagus was also observed (Figure 2B, white arrow). Subsequent history‐taking revealed that the patient had experienced progressive dysphagia for 2 years. Following barium esophageal studies and an upper gastrointestinal endoscopy, a diagnosis of achalasia and aspiration pneumonia was established. The patient was treated with intravenous antibiotics, and his symptoms improved. He was referred to a university hospital for a peroral endoscopic myotomy. Achalasia often manifests with dysphagia. However, up to 40% of patients report daily respiratory symptoms.^1^ Recurrent aspiration explains these symptoms, and even in some cases, it leads to aspiration pneumonia. On chest radiographs, patients with achalasia may have mediastinal enlargement, masses adjacent to the mediastinum, air‐fluid levels, and absent gastric air bubble. However, these patients often appear normal, especially in the early stages of the disease.^2^ In most of previous case reports, chest radiographs in patients with pneumonia due to achalasia showed findings suggestive of achalasia.^3^ However, as in the present case, even if chest radiographs show no evidence of achalasia, its presence may be detected on additional chest CT scans. Clinicians should be aware that gastrointestinal obstruction, especially due to achalasia, is one of the causes of aspiration pneumonia in young patients and consider performing additional CT scan imaging.

**FIGURE 1:**
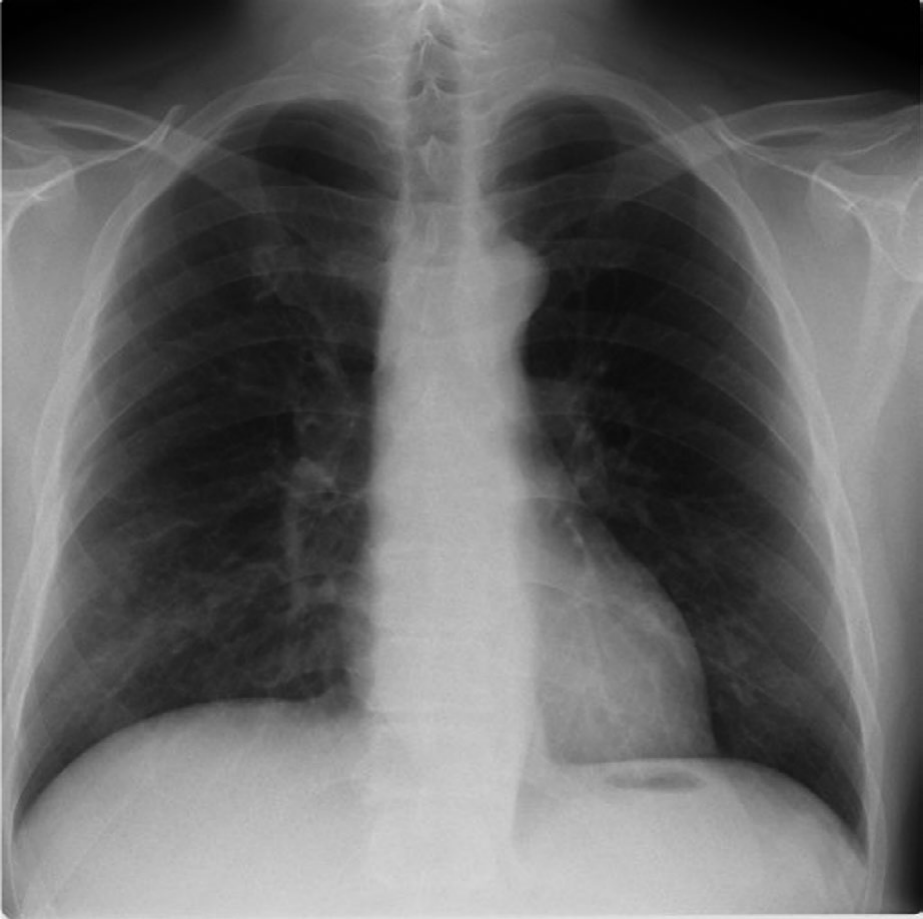
A chest radiograph only showing bilateral ground‐glass shadows

**FIGURE 2:**
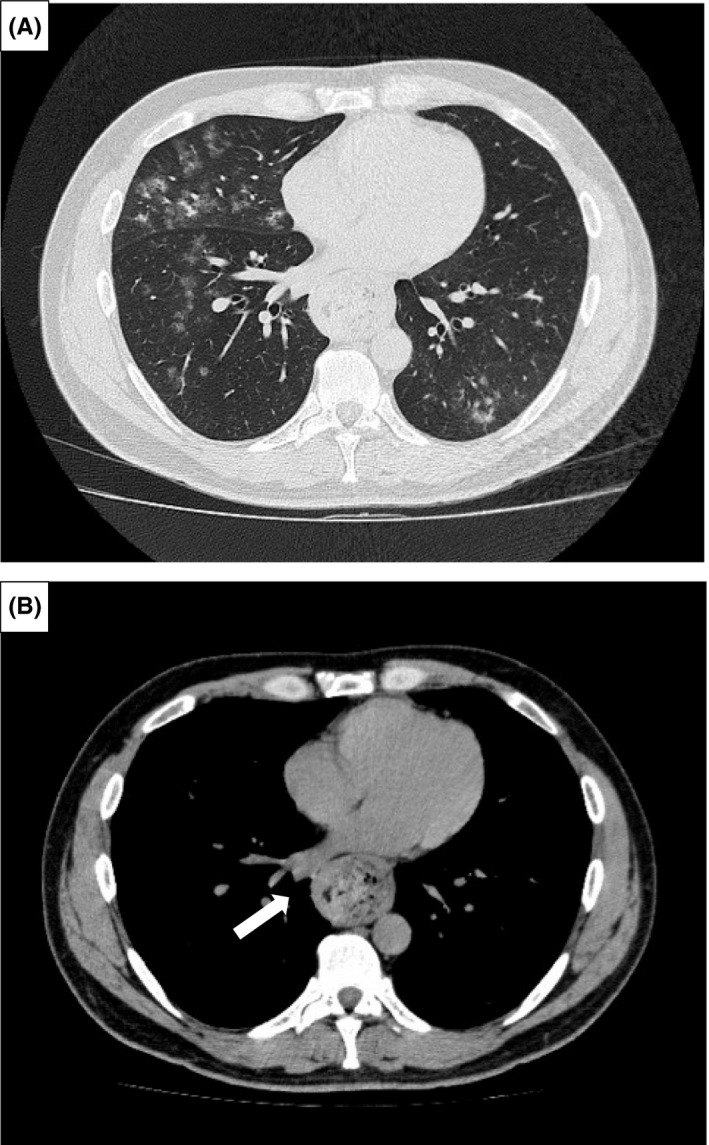
Axial CT scans of the chest (A: lung window; B: mediastinal window) showing small nodular shadow in both lungs and dilation of a food‐filled esophagus

## CONFLICT OF INTEREST

The authors declare no relevant conflict of interest that may arise from the conduct of this study or the preparation of this manuscript.
